# Decline of the boreal willow grouse (*Lagopus lagopus*) has been accelerated by more frequent snow-free springs

**DOI:** 10.1038/s41598-020-63993-7

**Published:** 2020-04-24

**Authors:** Markus Melin, Lauri Mehtätalo, Pekka Helle, Katja Ikonen, Tuula Packalen

**Affiliations:** 1 Natural Resources Institute Finland, Yliopistokatu 6b, 80100 Joensuu, Finland; 2 University of Eastern Finland, School of Computing, P.O. Box 111, 80101 Joensuu, Finland; 3 Natural Resources Institute Finland, Paavo Havaksentie 3, 90570 Oulu, Finland

**Keywords:** Climate-change ecology, Ecology

## Abstract

Climate change has influenced a range of species across the globe. Yet, to state a noted decline in the abundance of a given species as a consequence of a specific environmental change, for instance, spatially explicit long-term data are a prerequisite. This study assessed the extent to which prolonged snow-free periods in autumn and spring have contributed to the decline of the willow grouse, the only forest grouse changing into a white winter plumage. Time-series data of willow grouse numbers from summer surveys across the study area were integrated with local data on weather (snow cover), mammalian predator abundance and hunting intensity. Modelling was conducted with a hierarchical Bayesian Poisson model, acknowledging year-, area- and location-specific variability. The results show that while willow grouse numbers had decreased continuously across the study landscapes, the decrease was accelerated at the sites where, and during the years when the preceding April was the most snow-free. This indicates a mismatch between the change into a white winter plumage and the presence of snow, turning the bird into an ill-camouflaged prey. The results thus also confirm past hypotheses where local declines of the species have been attributed to prolonged snow-free periods. Across our study area, autumns and springs have become more snow-free, and the trend has been predicted to continue. Thus, in addition to conservation actions, the future of a species such as the willow grouse is also dependent on its ability to adapt to the changed environmental conditions.

## Introduction

Trends in global climate change are already affecting species and ecosystems^1–4^. Simultaneously, species and ecosystems are being affected by weather fluctuations within extreme values as well as by direct anthropogenic actions^5–7^. Even if the effects of normal weather anomalies, anthropogenic actions and climate change may be hard to disentangle, it is clear that they can exacerbate one another. Further, even when the mechanisms of species’ and ecosystem’s resilience towards environmental changes are well known, the impacts of abrupt changes in weather patterns may be too sudden for some species to adapt to. Concrete examples of such events are observable in the northern hemisphere where changing and diminishing snow covers have altered seasonal characteristics of entire landscapes^8^, and so affected the local flora and fauna^9,10^. The effect can be more serious the more dependent the species is on snow, and this seems to be the case with a boreal bird species that once was abundant all across Finland: the willow grouse (*Lagopus lagopus*).

Finland hosts willow grouse in two distinct populations, occupying different habitats: the northern populations occur in the areas of northern Finland with practically no forest cover, while the southern populations occur solely in forested areas, where they are typically found close to open mires and bogs within heavily forested landscapes. From now on, the term ‘willow grouse’ refers to the non-tundra populations occurring within the forested landscapes. The numbers of the tundra population have been comparably stable compared to those of the forest populations, which in the south, saw heavy, apparently warming-induced declines already in the 1930s. These were followed by partial revivals, but since the 1960s, the populations across southern and central Finland have been declining rather continuously^11–13^. To a large extent, this latter, more widespread decline has been attributed to the draining and afforestation of peatlands (mires, bogs) -widespread across Finland between the 1950s and the 1980s, affecting up to 80–90% of the peatlands in areas of Southern and Central Finland^14^. The drainage activities affected the reproductive output of all grouse species negatively^15^, but for the willow grouse the drainage has been attributed as one of the major drivers of its decline in southern parts of the country^11,16^.

Eventually, the loss of the natural mire habitats was seen as becoming too widespread, and since the 1990s, draining of new peatland areas has been virtually non-existent. In addition, campaigns to restore the peatland areas back to their original state have been conducted in growing numbers. Until 2013, some 20,000 hectares of drained peatlands have already been restored, with an additional 18,000 hectares in need^17^. For the declining willow grouse, additional conservation measures have also been taken: the species has been restricted or completely banned from hunting in central and southern Finland since the early 2000s. Shortly before this, it was discussed^18^ that the future of the willow grouse seemed positive, as habitat quality was likely to stay at least at the same level (due to the stopping of drainage activities and the promotion of biodiversity also within forestry). However, the same author also pointed out that the milder winters and the endured snow-free periods might pose a further threat, as was seen in the past, prior to the drainage activities. Indeed, when viewed today, the continuing decline of the willow grouse populations requires research on the extent to which the known decrease in snow covers in the boreal landscapes has affected it.

Snow is an especially crucial factor as the willow grouse, unlike the other forest grouse species in Finland has a white winter plumage. Ideally, the plumage is changed in synchrony with the arrival of the permanent snow cover in autumn as well as its disappearance in spring, and it has been suggested that its main function in the boreal zone is to provide camouflage^19^. To a strong degree, the changing of plumage is controlled by the amount of light, which means that the process is not in complete synchrony with possible changes in snow cover, and Finnish winters have seen decreased snow covers as autumns and springs have become warmer^19–21^. Therefore, the evolutionary response to snow could turn the willow grouse into an ill-camouflaged beacon against a dark ground – and as with the other forest grouse species, the main cause of willow grouse mortality, regardless of human hunting, is caused by predation by raptors, the goshawk (*Accipiter gentilis*) in particular^22–24^. Past studies have confirmed that a white willow grouse is further vulnerable to goshawk predation during autumns and especially springs^25–27^, but so far the potential declining effect of this phenomenon has neither been quantified nor assessed at population level. In this light, no conservation action can guarantee the survival of the forest willow grouse if, indeed, the decline from the past 20 years is attributed also to the more frequent snow-free autumns and springs. This is a highly topical question, since the boreal regions (including Finland) have warmed more rapidly than the other parts of the world with profound effects on snow covers^10,20,21,28^.

This study combines 21 years of willow grouse census data with data on predator abundance, hunting and local data on daily snow depth measurements. The aim of the paper is to assess the extent to which prolonged snow-free periods in autumn and spring explain or contribute to the observed decline in willow grouse populations on the consecutive summer. The results are viewed in light of the recent predictions on how climate change may affect boreal winters and what this means for the future of the species, even with heavy conservation efforts.

## Materials and Methods

### Willow grouse data

Data on willow grouse abundance were provided by the Natural Resources Institute Finland (LUKE). The data were obtained from the Wildlife Triangle Censuses, a wildlife population monitoring program that has been active in Finland since 1989. Altogether, there are over 1,500 monitoring triangles located across Finland, and around 900 of these are surveyed annually. The ‘triangle’ itself is an equilateral triangle with each side measuring 4 km, adding up to form a 12-km long permanent inventory route. The route is checked twice a year (winter and summer), with the main purpose of providing information on the status of wildlife populations^12^. The summer counting is done in late-July or early-August, specifically to estimate the grouse numbers and the success of grouse breeding prior to deciding the length of, and possible restrictions to, the autumn´s grouse hunting season. The route is surveyed by a three-person team (volunteers, local hunters), who walk the route and mark the locations of every grouse observed along the way (inside a 60-meter-wide belt). The data thus consists of observations based on grouse adults and broods, separated by species. In the winter survey, all animal tracks crossing the survey route are counted, and based on this, an abundance index is derived: number of crossings/24 h/10 km. For determining such an index, the survey is conducted with strict protocols: either the survey is made 1–2 days after a fresh snowfall, or alternatively, the route can be surveyed twice: all animal tracks are marked during the first survey, after which the second, proper survey and the counting of new tracks is conducted 1–2 days later. The wildlife triangle census was launched in 1989, and the data have been of crucial importance for a variety of wildlife- and grouse-related studies^29–31^. However, based on expert’s knowledge we only use data from the year 1996 onwards due to the data collection methods and protocols not being fully harmonized during the first survey years.

In this study, the winter data were used to gain information on the abundance of pine marten *(Martes martes*) and red fox *(Vulpes vulpes)*, opportunistic yet potential predators of willow grouse, and the summer data were used to gain information on willow grouse abundance. Willow grouse chicks were not included in the analysis, as their mortality would not be affected by autumn and spring-time snow conditions; they are born in June after which their mortality is primarily determined by post-hatching weather and nest predation. The data consists of 4,897 observations: 1,159 grouse cases (consisting of 2,088 individuals) and 3,738 no-grouse cases.

Data on human hunting were obtained also from LUKE. The hunting statistics have been collected with a harmonised method since 1996 and contain county-specific information on annual hunting bag by species. In most parts of our study area, willow grouse hunting was common in the early years of the study, while it has largely been non-existent since 2009. Yet, its inclusion was justified as this allowed to evaluate the effect of hunting in the decline.

### Study areas

**Figure 1:**
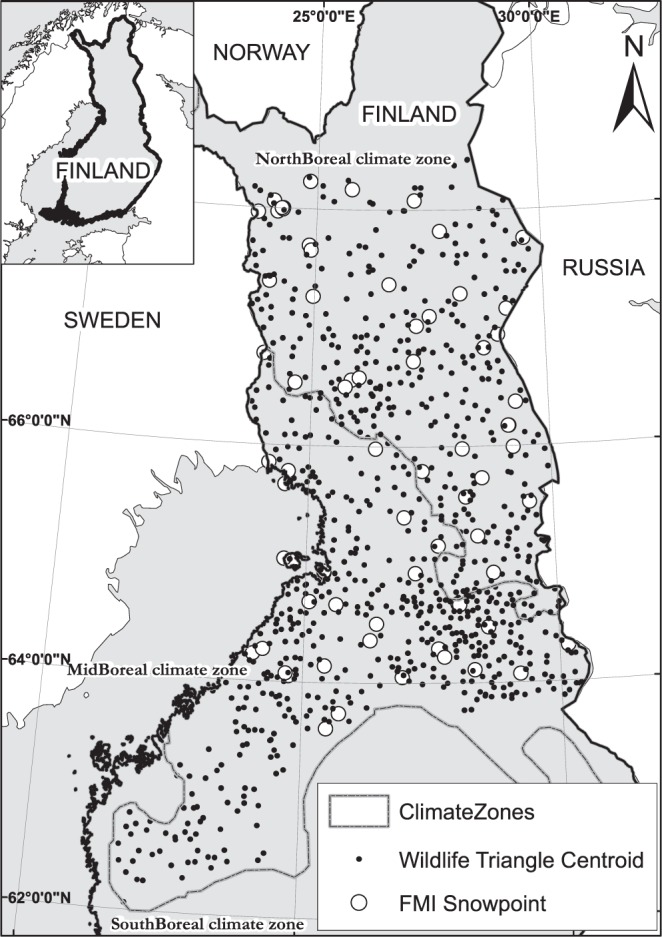
The study area and the distribution of wildlife triangles in Finland. The excluded area in northern Finland approximates the area where the forest willow grouse ceases to exist, and the populations are formed by the tundra willow grouse.

**Figure 2:**
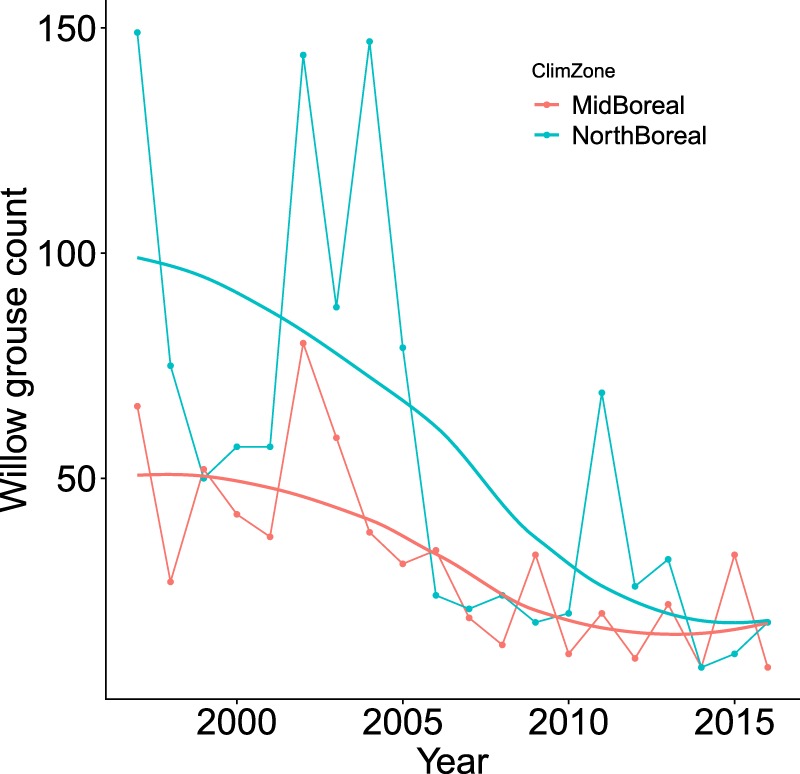
Number of observed willow grouses in the two climatic zones per year. The smoothed trendlines are for illustrative purpose only.

The study was conducted in areas where the willow grouse had been abundant enough since 1996 to assess any related patterns. Figure 1 shows a map of Finland highlighting the study area and the distribution of the wildlife triangles within it. The climatic zones in Fig. 1 are defined by the Finnish Meteorological Institute, and their delineation is based on rain- and snowfall as well as temperature, making them valid for this study. Here, the study area’s triangles were within two zones, *NorthBoreal* and *MidBoreal*. Altogether, 2,487 triangles were counted from the *MidBoreal* climatic zone, on average 124 per year (standard deviation 22). From *NorthBoreal*, 1980 triangles were counted in total, on average 99 per year (standard deviation 19). Figure 2 shows the average trend in the willow grouse populations as well as the number of counted triangles per year in the two climatic zones.

### Data on snow cover

**Figure 3:**
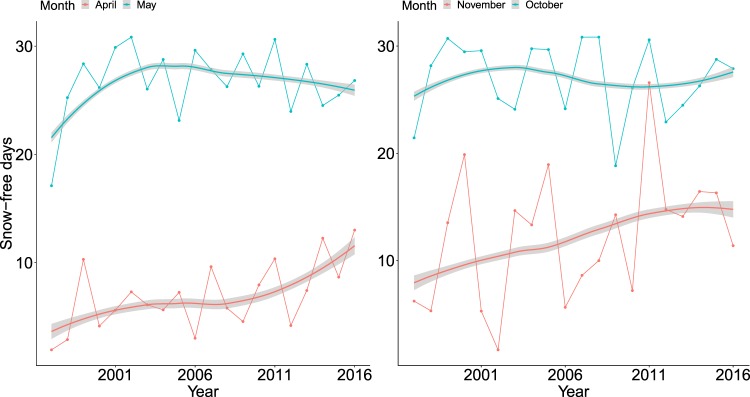
Average number of snow-free days in October, November, April and May in the study area between 1996 and 2016 (thin lines with points), and the smoothed average trend over the years (bold lines with grey polygon that depicts 95% confidence interval for the mean).

Snow data were gained from the Finnish Meteorological Institute (FMI), derived from snow depth measurement points located across Finland (Fig. 1). Each data point of the FMI stores daily snow depth measurements. Here, the months of focus were October, November, April and May, the months when the species changes into, and out of, the white winter plumage. Further, the FMI data showed that these were the months when snow typically arrived and disappeared in the study area, and these were also the periods that had changed in relation to the presence of snow (Fig. 3).

To estimate the local weather conditions at the location of each triangle, the weather data from the nearest FMI snow measurement point and weather station was associated with them. While it is clear that snow depths will vary significantly within a landscape, the mere ‘presence’ of snow does so in a lesser extent. Also, the FMI data points are placed on open locations, and open areas in a forested landscape are what the willow grouse favours as well. Furthermore, the zero-mean errors in the interpolated predictors would only add variability to the predictors, and so the only harm they can cause is in attenuating the regression coefficients towards zero – making the setting more conservative when it comes to under- or overestimating the results^32^. Now, each triangle (and for each year) gained the associated snow data from the snow depth measurement point that was closest to it. Next, monthly snow statistics were derived for each triangle (Table 1).

**Table 1:** The variables included in the selection process.

Variable	Explanation
*Density_Fox*	Fox density at the location of each wildlife triangle.
*Density_Marten*	Pine marten density at the location of each wildlife triangle.
*Hunting*	Annual willow grouse hunting bag.
SnowFreeDays*	Number of snow-free days in this month.
AvgSnowDepth*	Average snow depth of this month (cm).
MedianSnowDepth*	Median snow depth of this month (cm).
PlusDays*	Number of days in this month when temperatures were constantly above 0 degree Celcius.

^*^These variables were tested both from the seasons preceding the August bird census and the seasons two years earlier.

The snow- and weathervariables were calculated for all target months (October, November, April, May).

The selection of variables was based on prior expertise and knowledge about the ecology of the species in question. In general, one could have chosen hundreds of weather variables and input them in a machine learning framework, revealing cause-effect relationships majority of which would have had nothing to do with the ecology of the species or how it relates to the environment. We restricted the analysis to a limited number of well-justified variables to minimize the risk for false positive results (type I error). Here, it was known that the snow covers across the study area had diminished, and from earlier studies it was also hypothesized that the lack of snow during plumage changing will expose the willow grouse to increased raptor predation, which may drive their numbers down on a local scale^16,25–27^. The task of the modelling was also to assess whether these hypotheses were supported by our current data.

### Statistical analysis

Modelling between snow-related variables and willow grouse numbers was done using Generalized Linear Mixed Modeling (GLMM), applying the Poisson distribution with log link. The grouping of the data to different years and individual triangles was modelled by using random effects. As the clear decreasing trend in willow grouse numbers (Fig. 2) could be explained by any variable that has a trend over time, this was modelled (and included in the model) with a spline function. Next, the association between the predictors (Table 1) and willow grouse numbers was explored. That is, did any of the variables in Table 1 contribute to the decline of the willow grouse when acknowledging year- and triangle-specific variation? Variable selection for the modelling was done by forward selection, where the single most significant variable was first added to the model, and it was accompanied by another variable only if the new variable proved to be significant. The process was iterated until no more variables could be added.

The final model included only one significant (p < 0.05) variable: the number of snow-free days in April. Therefore, the final model assessing the effect that snow-free April days have on adult willow grouse in triangle *i* during year *j* was as follows:

$${y}_{ij} \sim Poisson({\mu }_{ij})$$

$$\log ({\mu }_{ij})={\beta }_{0}+f(y{r}_{j};{\boldsymbol{\gamma }})+{\beta }_{1}SF{4}_{ij-1}+{\beta }_{2}{N}_{i}+\ldots +{b}_{i}+{c}_{j}+{u}_{ij},$$

where $y{r}_{j}$ is time in years since 1996, $SF{4}_{ij-1}$ is the number of snow-free days from the preceding April, ${N}_{i}$ is the indicator variables for the northern zone, “…” stands for the other predictors (Table 1) that were finally dropped from the equation, ${b}_{i}$ and ${c}_{j}$ are normally distributed, zero-mean random effects for triangle and year, respectively, and ${u}_{ij}$ is the vector of random observation errors, which takes into account the overdispersion in the data. Term *f*(*yr*_*j*_;***γ***) is the nonlinear time trend, which was modelled by using restricted cubic splines using four knots placed at years 1999, 2003, 2007 and 2015, and the regression coefficients ***γ*** for the spline components. The spline retains the model as linear, where the predictors are $y{r}_{j}$ and two truncated power transformations of it^33^.

It might be possible that the effect of variable *SF*4_*ij*−1_ would be an artefact, which actually explains the effect of small-scale variability in the landscape on the grouse abundance instead of the effect of snow conditions of the previous winter. If that would be the case, then the two-years lagged variables *SF*4_*ij*−2_ should explain the variability in grouse abundance equally well as $SF{4}_{ij-1}$. Therefore, the model was fitted both by using the one-year lagged and two-year lagged variables as the predictors.

To double-check the conclusions of the modelling, the model was fitted using traditional likelihood-based methods as implemented in the R-package *lmer*, but also in the Bayesian framework using the Markov Chain Monte Carlo method as implemented in the R package *MCMCglmm*^34^. The default prior distributions of the package (normal for regression coefficients and inverse Wishart for variances) were used. The trace plots showed good mixing of the MCMC chains. However, as both models led to similar conclusions with only marginal differences in parameter estimates, only the results based on the MCMC method are reported. Figures were drawn using the packages *ggplot2*^36^ and *cowplot*^37^. In general, the GLMM approach was chosen as it allowed acknowledging the dependence caused by the grouping of the data to measurements within triangles^35^.

## Results

### Drivers of willow grouse numbers

The single most significant driver of willow grouse numbers (and the only significant one) was the amount of snow-free days in the preceding April (p-value = 0.002, Bonferroni-adjusted p-value = 0.014 based on the 7 potential evaluated predictors). The other tested variables (Table 1) did not have a significant effect on willow grouse numbers over the normal variation caused by year- and area-specific differences.

Results from the modelling showed that each additional snow-free day in April caused an additional 3.1% decline in willow grouse numbers (Table 2 – *SnowFreeDays_APR*). From Fig. 3 one sees that the number of snow-free days has been increasing. Therefore, an April with five snow-free days results in ca. 14% less observed grouse.

**Table 2:** The final model showing the effect of the strongest predictor, snow-free days in the preceding April, on willow grouse numbers.

Variable	Posterior mean	Lower 95%	Upper 95%	p
Fixed effects:				
*Intercept (*${\beta }_{0}$*)*	−2.99	−3.55	−2.33	
*Year (*${\gamma }_{1})$	0.07	−0.07	0.21	
*spline component 1(*${\gamma }_{2})$	−0.004	−0.07	−0.001	
*spline component 2(*${\gamma }_{3})$	0.01	0.002	0.02	
*ClimZone_N (*${\beta }_{2})$	0.87	0.55	1.19	<0.001
*SnowFreeDays_APR (*${\beta }_{1})$	−0.031	−0.05	−0.01	0.002
**Variances of random effects:**				
*Year -* $var\,({c}_{j})$	0.26^2^	0.001^2^	0.41^2^	
*Triangle -* $var\,({b}_{i})$	1.01^2^	0.82^2^	1.19^2^	
**Overdispersion parameter**				
$var\,({u}_{ij})$	1.83^2^	1.68^2^	1.99^2^	

**Figure 4:**
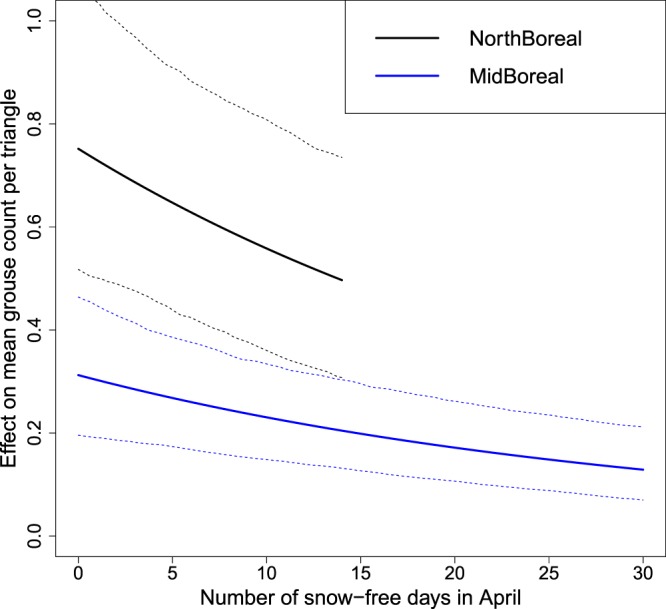
The predicted effect of snow-free April days on willow grouse numbers in the different climatic zones based on the estimated model (Table 2). The dashed lines show the Bayesian 95% credible intervals for the mean. The effect of snow-free April is the same in both regions, even though the lines differ because of the scaling of the y-axis. One additional snow-free day decreases the population by 3.1%, meaning that a year with 5 snow-free April days had ca. 14% less willow grouse than a year with fully snow-covered April.

Replacing the significant one-year lagged predictor with the two-years lagged predictor led to a model where the snow-free April days had no effect. Therefore, it was the April instantly before the August surveys that had the significant decreasing effect. If predicted across the range of snow-free days in April, the negative effect is clear (Fig. 4).

## Discussion

In assessing the decline of the willow grouse in the 1930s, Kalela^26^ stated that “*of the boreal species that have, evidently due to climatic changes, become more infrequent in southern Finland, the best known example is the willow grouse*”. Here, we examined whether local weather anomalies, more frequent snow-free seasons in particular, have played a role in the decline of the forest willow grouse populations also in more northern areas of Finland, where the effects of climate change in relation to temperature and snow have been, and are predicted to be, stronger than the global average^20,21,28^. In general, the topic of camouflage mismatch in causing excess predation to arctic species has been recently discussed and studied in increasing amounts due to the rapid warming and diminished snow covers in the boreal and arctic zones^10,19^.

It is not straightforward, however, to state an observed pattern as a pure consequence of climate change or even the more frequent snow-free days, as a multitude of factors may be, and often are, involved. Indeed, cases where a species itself would not react to e.g. a warming climate, but is then influenced by competitors, predators or parasites that gain from the warming are anything but unheard of^38–41^. Hof *et al*.^42^ linked the decline of the arctic fox (*Alopex lagopus*) not directly to a warming climate, but to the fact that the warming climate caused declines in the arctic fox’s main prey, while aiding the range expansion of a stronger competitor, the red fox (*Vulpes vulpes*). In Canada, it has been suggested that conservation plans for the woodland caribou (*Rangifer tarandus caribou*) should account for the climate-induced range expansion of the moose (*Alces alces*)^43^.

Here, the research setting and the long-term data allowed to include year- and area-specific variation and to exclude some of the potential alternative factors. Human hunting on willow grouse in our study area was minimal and in most parts, non-existent since 2009. The tested mammalian predators, red fox and pine marten are generally considered only as opportunistic predators; in a mortality study in Sweden, mammalian predators were responsible for zero willow grouse deaths during winter and spring^23^. Despite the effects of hunting and mammalian predation being insignificant, the decline cannot be attributed to climatic changes alone. Indeed, field studies done in Sweden found raptors to be the most frequent cause of willow grouse mortality, also regardless of human hunting^23^. In Finland, the predation pressure by raptors has also been noted for other forest grouse species^22,24^, but in parts of our study area, the willow grouse was noted to be the most favoured prey among the different grouse species^27^. Furthermore, it is the only forest grouse changing into a white plumage, which in the event of a mismatch between plumage-changing and absence of snow would ruin its camouflage against a predator that is favouring it already. The recent Finnish Breeding Bird Atlas does not show a decline in the abundance of the birds of prey in the study region^44^.

The periods when the effect of snow-free ground was assessed were the months when snow most typically arrives in (October and November) and disappears (April and May) from the study area. In addition, these are the months when the changing into and out of the winter plumage takes place. Snow-free landscape in autumn or spring is not only harmful for the willow grouse, as in such cases, the species would be able to use the most preferred source of food (berry plants) for a more prolonged period^45,46^. Therefore, there should have been no other obvious reasons (than the mismatched winter plumage) for the populations decline due to snow-free springs. Here, we hypothesised that a white winter plumage in a snow-free landscape leads to increased predation pressure from birds of prey, and we were not the first ones to make the connection: in the 1930s, well before the drainage activities, the species declined sharply in southern and central Finland. The reasons behind this decline were linked to warmer seasons and prolonged snow-free periods, which were deemed to be potentially unsuitable for the species altogether, but at least exposing it to excess goshawk predation^16,25,26^. These southern parts of Finland were then later heavily affected by the drainage activities. In assessing willow grouse populations in Ostrobothnia region between 1965 and 1988 (southern extent of our study area), Tornberg and Sulkava^27^ discussed that snow-free seasons, springs in particular, caused a plumage mismatch “*making the willow grouse exceptionally vulnerable to goshawk predation*”.

In our results, the fact that the lack of snow in the often snow-free May (Fig. 3) did not have a negative effect suggests that the camouflaging summer plumage has already been changed and the species is better equipped against avian predation. Indeed, the increase in the number of snow-free days in April was the single most negative variable affecting willow grouse population numbers (Table 2 **–** Fig. 4**)**. According to the parameter estimates, each snow-free day in April caused a decline of 3.1% (95% confidence interval 1–5%) in the population. This alone does not sound dramatic, but as one observes the effect across the range of snow-free days in April (Fig. 4) and acknowledges the trend in snow-free April days (Fig. 3) and the predicted climatic changes in Finland^20,21^, the situation becomes more alarming.

The use of two-year lagged variable (number of snow-free days in April two year before) could not explain the variability in species abundance, when it was used instead of the one-year lagged predictor. This supports our conclusion that the decrease is associated with the mortality in the snow-free period of the previous April, instead of some local properties that are associated with the local average of the snow-free days: if the phenomenon was driven by variables of landscape structure, the effect would not have varied between two consecutive years – the land does not change its properties that fast, weather does. We also fitted a model where both the one-and two-year lagged snow-free days in April were included in the same model as predictors, but only the one-year lagged predictor was significant. Of course, the snow-cover induced local decrease in the abundance might be visible in the data also after two years. However, the local differences caused by local snow conditions of a given year will gradually disappear because the birds can move, and the *Lagopus* -species are known to be very capable of doing so^23,47–49^. Therefore, these long-term effects (for lag 2 and higher) are most likely modelled by the time trend component of our model.

This study, as its predecessors^11,15,26,27^, was made possible by long-term monitoring data on wildlife populations as well as on variables of weather. The value of these kinds of datasets is extremely high if we are to properly assess the impacts that the occurred climatic changes have had on our wildlife populations. Indeed, if one would have observed merely the average 20-year trend in the willow grouse numbers, the association of the decline to any given variable would have been possible, but any statements about the causal effect would have been very questionable. Our data allowed an analysis of the local annual anomalies from the overall trend in the abundance of the species, and explaining the deviations by local weather and snow cover conditions from the two preceding winters. Previous studies were used to construct hypotheses about the effects of snow cover, mammal predation and hunting pressure on the abundance. These hypotheses about the effect of snow-free ground were supported by our data, whereas the effects of mammal predation and hunting were so weak that they did not become statistically significant. Of course, it is always possible that the observed effect in an observational data set is caused by confounding factors instead of the true causal relationship. We protected ourselves against such misinterpretation by (1) restricting the analysis to only few carefully chosen potential predictors that are supported by the previous studies, (2) adjusting the results for the multiple tests, and (3) exploring whether the snow conditions of the previous spring could be replaced by snow conditions two years before the measurements. We conclude that, most likely, there is a causal relationship between the April snow conditions and willow grouse abundance of the following summer, and the relationship is caused by increased exposure of the white birds in the snow-free environment to their most significant predators (the birds of prey). After all, the white winter plumage is primarily meant to camouflage arctic species against predation^19^. Of course, a controlled experiment would be a better tool to analyse the causal relationship, but organizing such for the current research question would be practically impossible and unethical. Therefore, the current results shows, in our opinion, the strongest evidence from the best available data sets on the effect of diminishing snow covers on the abundance of willow grouse – a phenomenon discussed and assessed also in the past. The hypothesis is supported also by research done in North America and Italian Alps on other seasonally colour coating species such as the snow-show hare (*Lepus americanus*)^50^ or a close relative of the willow grouse, the willow ptarmigan (*Lagopus muta*)^51^.

Past studies have shown that the willow grouse can revive given the right circumstances. After the decline of the 1930s, the late 1940s saw the willow grouse becoming abundant in parts of southern Finland again (though not as abundant as it was prior to the decline)^52^. Given that we know now the devastating role of drainage, conservation has still a good chance for success, given the species’ high reproductive output. Further research should thus be made on the additional drivers behind the population decline as these can turn out to be variables that, unlike weather, could be controlled by human actions. In Finland, severe conservation measures were started in the late 1990s, with the aim to restore peatland areas in central and southern Finland. These involved the filling of the ditches at some of the drained peatlands, for instance, which would eventually be beneficial also for the willow grouse and thus aid the return of the species. Field studies have confirmed that restored peatlands have been accepted as habitats by the willow grouse^53^. Yet, an equally topical question is also the ability of the species to track and adapt to the changed, and changing, snow conditions under the current climatic scenarios for boreal winters^21^. Indeed, while the species can decline even without a climatic effect^52^, a phenomenon such as warming or the prolonged snow-free periods has never been linked to an increase in willow grouse populations, but evidence from its declining effect is existing – as shown in this paper, in addition to the past papers. The current climatic predictions point to significantly diminishing snow covers in southern and central Finland^54^ – a change that is evident from past data as well. It seems that in the worst cases, the strongly light-controlled trigger that controls the camouflage of the willow grouse and other seasonally coat coloured arctic species^19^ is not in synchrony with what the species is trying to camouflage in, and these kinds of conditions are to become more frequent^51^.

## Data Availability

The datasets generated during and/or analysed during the current study are not publicly available due to nature of the data: they are data of the official authority and not to be shared publicly. However, the compiled datasets created for this study are available from the corresponding author on reasonable request. Also, information on the willow grouse population trends can be seen from the wildlife triangle survey’s web-pages at: https://www.riistakolmiot.fi/animal/riekko-lagopus-lagopus/ (auto-translation needed).
